# Three-dimensional induction of dorsal, intermediate and ventral spinal cord tissues from human pluripotent stem cells

**DOI:** 10.1242/dev.162214

**Published:** 2018-07-30

**Authors:** Takenori Ogura, Hideya Sakaguchi, Susumu Miyamoto, Jun Takahashi

**Affiliations:** 1 Department of Clinical Application, Center for iPS Cell Research and Application, Kyoto University, 606-8507 Kyoto, Japan; 2 Department of Neurosurgery, Kyoto University Graduate School of Medicine, 606-8507 Kyoto, Japan

**Keywords:** Human pluripotent stem cell, Spinal cord tissue, Development, Spinal interneurons, Spinal motor neurons

## Abstract

The spinal cord contains more than 20 distinct subclasses of neurons that form well-organized neural circuits capable of sensing the environment and generating motor behavior. Although recent studies have described the efficient *in vitro* generation of spinal motor neurons, the induction of the spinal cord as a whole tissue has not been achieved. In the present study, we demonstrate three-dimensional (3D) induction of dorsal spinal cord-like tissues from human pluripotent stem cells. Our 3D spinal cord induction (3-DiSC) condition recapitulates patterning of the developing dorsal spinal cord and enables the generation of four types of dorsal interneuron marker-positive cell populations. By activating Shh signaling, intermediate and ventral spinal cord-like tissues are successfully induced. After dissociation of these tissues, somatosensory neurons and spinal motor neurons are detected and express neurotransmitters in an *in vivo* manner. Our approach provides a useful experimental tool for the analysis of human spinal cord development and will contribute to research on the formation and organization of the spinal cord, and its application to regenerative medicine.

## Abstract (precis)

**Summary:** The three-dimensional induction of dorsal, intermediate and ventral spinal cord-like tissues from human pluripotent stem cells described provides a useful experimental tool for studying human spinal cord development.

## INTRODUCTION

Spinal cord tissue consists of well-organized neural circuits that process large amounts of information for the organism to sense its environment and generate motor behavior. During early embryonic development, spinal cord tissues are initially formed from a sheet-like structure termed the neural plate. The neural plate bends towards the dorsal side of the embryo and forms a tubular structure: the neural tube (Novitch et al., 2003). During dorsal-ventral patterning of the neural tube, more than 20 distinct classes of neurons are generated (Alaynick et al., 2011; Lu et al., 2015). Two organizing centers play important roles in determining the characteristics of neuronal progenitor cells during spinal cord formation. One organizing center is the roof plate (RP), which is located dorsally and induces dorsal progenitor domains by producing bone morphogenetic proteins (BMPs) and Wnts (Timmer et al., 2002; Chizhikov and Millen, 2004b). The other organizing center is the floor plate (FP), which is located ventrally and induces ventral progenitor domains by producing sonic hedgehog (Shh) (Jessell, 2000). With the help of these two morphogen-producing organizing centers, six discrete dorsal progenitor domains and five ventral progenitor domains are generated along the dorso-ventral axis, and various subclasses of spinal interneurons and motor neurons are generated from the patterned progenitor domains (Goulding, 2009).

Recently, much attention has been paid to the efficient generation of spinal motor neurons from human pluripotent stem cells (hPSCs) (Amoroso et al., 2013; Du et al., 2015; Maury et al., 2015). Several reports describe the induction of patterned neural tube-like structures using mouse embryonic stem cells (ESCs); however, the formation of a three-dimensional (3D) structure of spinal cord tissues that includes the patterned induction of various types of spinal neurons has not been described (Peljto et al., 2010; Meinhardt et al., 2014; Ranga et al., 2016). Because the spinal cord works as an independent functional unit, it is necessary to induce not only single spinal motor neurons, but also total spinal cord tissue if one wants to analyze the spinal cord as a completely functional organ system. The derivation of a 3D spinal cord equivalent would thus allow better understanding of spinal cord development and contribute to the generation of valid *in vitro* disease models.

To overcome the aforementioned limitations, we sought to achieve the 3D induction of spinal cord tissues from hPSCs. By modifying a previously described protocol for spinal motor neurons, we first established a new differentiation condition for dorsal spinal cord-like tissue induction, making it possible to generate four distinct dorsal interneuron marker-positive cell populations. The character of these tissues could be dorsalized by BMP4 treatment. Activation of Shh signaling, on the other hand, led to the successful formation of intermediate and ventral spinal cord-like tissues. Furthermore, these hPSC-derived *in vitro* tissues could generate several types of spinal neurons that mimic patterns of the developing spinal cord *in vivo*. Our hPSC-based *in vitro* induction condition recapitulates the *in vivo* developmental process of spinal cord formation and thus represents a useful tool for studying human spinal cord-related diseases and potentially for drug discovery and regenerative medicine.

## RESULTS

### Induction of patterned dorsal spinal cord-like tissues from hPSCs

To induce spinal cord tissues, we first induced spinal motor neurons following a previously described protocol (referred to as the SMN protocol). Because our goal was 3D structure formation, we used a SFEBq-based approach, which has previously been shown to efficiently induce 3D neural tissues *in vitro* (Maury et al., 2015; Eiraku et al., 2008). A feeder-free human induced pluripotent cell (iPSC) line (1039A1) was dissociated to single cells and 9000 cells were seeded into each well of U-bottomed 96-well plates with low-adhesion coating. The cells reaggregated quickly to form a single embryoid body per well and were cultured using the SMN protocol (Fig. S1A). On culture day 15, a large number of Olig2^+^/Nkx6.1^+^ spinal motor neuron progenitor cells were identified (Fig. S1B). On day 24, the generation of Hb9^+^/Islet1^+^ motor neuron precursors was confirmed (Fig. S1C). To evaluate the continuous epithelial structure in the aggregates, we examined the expression pattern of N-cadherin. Although PAX6^+^/N-cadherin^+^ neural progenitors were observed in the aggregates on day 15, the expression of N-cadherin was observed only in a discontinuous manner (Fig. S1D-F). This result demonstrated that proper 3D epithelial structure formation could not be obtained under the SMN condition.

**Fig. 1.**
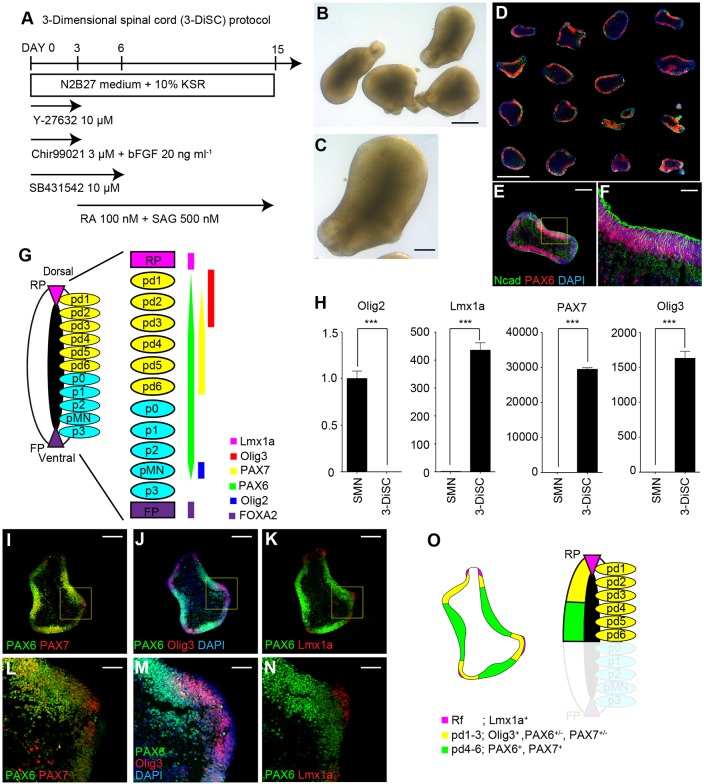
**Induction of patterned dorsal spinal cord-like tissues from hPSCs.** (A) Schematic of the differentiation protocol for spinal cord tissues (the 3-DiSC condition). (B,C) Phase-contrast images of the aggregates on day 15. (D-F) A PAX6^+^/N-cadherin^+^ continuous neuroepithelial structure was efficiently formed on day 15. (G) Schematic showing the expression pattern of progenitor domain markers in the developing spinal cord. (H) qPCR analysis on day 15 showing the relative expression of progenitor domain markers under the 3-DiSC condition compared with the SMN condition (***P<0.001, *n*=6 total RNA samples from three independent culture experiments, unpaired *t*-test with Welch's correction, two-tailed). Data are mean±s.e.m. (I-K) Immunostaining of serial section of an aggregate on day 15 showed the generation of dorsal spinal cord-like tissues. (I,L) The PAX6^+^ continuous epithelium co-expressed PAX7. (J,M,K,N) Several regions in the Pax6^+^ neuroepithelium showed the expression of Olig3, and Lmx1a^+^ domains were induced adjacent to Pax6^+^/Olig3^+^ domains. (O) Schematic summary showing the dorsal spinal cord-like tissues induced by the 3-DiSC condition. Scale bars: 1000 μm in D; 500 μm in B; 200 μm in C,E,I-K; 50 μm in F,L-N.

To form the 3D structure of spinal cord tissues, we tried to establish a new condition. Many small molecules were included in the original SMN protocol to modulate signaling pathways and restrict the generated cell populations to spinal motor neurons. We hypothesized that eliminating some of the small molecules would induce wider regions of the spinal cord. We tested the modified condition under which LDN193189 (BMP inhibitor) and SAG (smoothened agonist) were removed with supplementation of basic differentiation medium [referred to as the 3-dimensional spinal cord (3-DiSC) condition] (Fig. 1A). Under this condition, continuous epithelial structures were observed in the aggregates on day 15 (Fig. 1B,C). Immunohistochemistry (IHC) analysis showed the efficient formation of PAX6^+^/N-cadherin^+^ continuous neuroepithelial structures (Fig. 1D-F).

Next, we examined the character of the progenitors in the formed continuous neuroepithelium. Because each progenitor domain can be distinguished by the expression pattern of distinct transcription factors, we first checked the expression of progenitor domain markers using quantitative PCR (qPCR) (Fig. 1G) (Lu et al., 2015). Under the SMN protocol, the expression of Olig2 (marker for pMN) was evident, while the expression of markers for dorsal domains such as Lmx1a (a marker for roof plate), Olig3 [a marker for progenitor domains 1-3 (pd1-3)] and PAX7 (a marker for dorsal progenitor domains) was not (Fig. 1H). Conversely, under the 3-DiSC condition, the expression of Olig2 was repressed, while the expression of the aforementioned dorsal domain markers was strongly upregulated.

We further analyzed and validated the properties of hPSC-derived 3D continuous epithelial structures by IHC. Continuous epithelial structures mainly expressed PAX6 and PAX7, suggesting the generation of dorsal progenitor domains (Fig. 1I,L). Several regions in the Pax6^+^ neuroepithelium showed the expression of Olig3 (Fig. 1J,M). Adjacent to the Pax6^+^ Olig3^+^ regions, the expression of Lmx1a could be observed, suggesting the generation of Lmx1a^+^ roof plate next to Olig3^+^ pd1-3 domains (Fig. 1K,N). The expression pattern of these markers was similar to the developing embryonic dorsal spinal cord (Fig. 1O). The formation of the structures was observed in 93.4% of the analyzed aggregates in four independent experiments. We obtained similar results for other human iPSC lines and a human ESC line (Fig. S2A-P).

Taken together, our 3-DiSC condition enabled the efficient *in vitro* induction of continuous epithelial structures that express several dorsal progenitor domain markers, which is similar to the developing embryonic dorsal spinal cord.

### Neural fate commitment in early phase under 3-DiSC condition and the emergence of roof plate-like structures

**Fig. 2.**
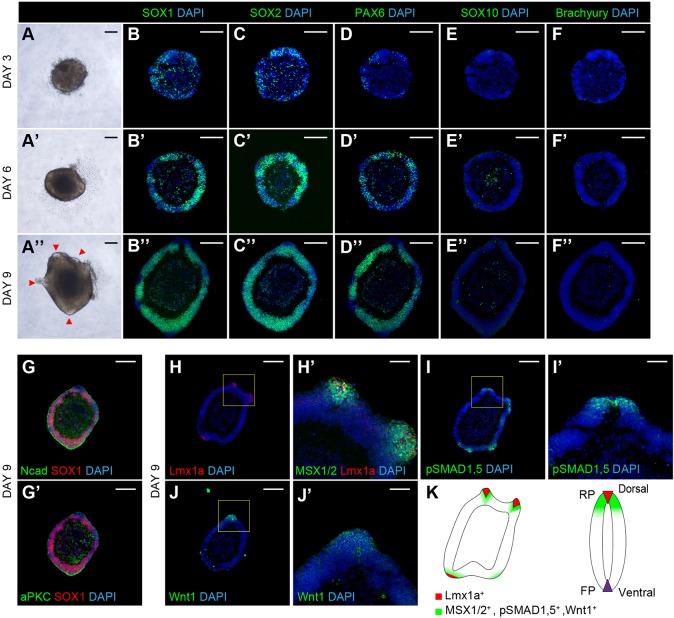
**Neural fate commitment in early phase under the 3-DiSC condition and the emergence of roof plate-like structures.** (A-A″) Phase-contrast images during the early phase of the culture period showing the generation of the continuous epithelial structure and several protrusions. Small protrusions were formed on the epithelial structure from day 9 (red arrowheads in A″). (B-F″) Immunostaining of aggregates cultured under the 3-DiSC condition for SOX1, SOX2, PAX6, SOX10 and brachyury. (B-B″,C-C″,D-D″) A SOX1^+^ SOX2^+^ PAX6^+^ continuous neuroepithelial structure was formed for the first 6-9 days. (E-E″) A small number of SOX10^+^ cells were observed, and the cells were confined to the inner part of the aggregate. (F-F″) Brachyury^+^ cells were rarely detected. (G,G′) N-cadherin and aPKC were mainly expressed in the outer side of the epithelium. (H,H′) Immunostaining for Lmx1a and MSX1/2. (H) The expression of Lmx1a was observed on the protrusion. (H′) MSX1/2 was expressed in a wider region than the Lmx1a^+^ region. (I,I′) Immunostaining for phospho-Smad 1/5. The expression of phospho-Smad 1/5 correlated with the expression of MSX1/2. (J,J′) Immunostaining for Wnt1. The expression of Wnt1 was also detected around the protrusion structure. (K) Schematic summary showing the expression pattern of BMP and Wnt signaling-related markers. Scale bars: 200 μm in A-H,I,J; 50 μm in H′,I′,J′.

Next, we examined how patterned dorsal progenitor domains were formed from hPSC aggregates. We first investigated the morphological change and neural marker expression pattern of the aggregates. After hPSCs were seeded into each well of a U-bottomed 96-well plate, a cell aggregate became obvious from 10 h after the start of differentiation (Movie 1). Imaging also showed that a small number of cells surrounded and were not integrated into the aggregate. On day 3, round-shape aggregates were formed (Fig. 2A); on day 6, a continuous epithelial structure was obviously generated; and on day 9, several small protrusions were formed in the aggregates (Fig. 2A′,A″). The expression of SOX1/SOX2 was broadly seen in the aggregates at day 3, but it was restricted to the neuroepithelial structure on day 6 (Fig. 2B,B′,C,C′). The expression of PAX6 was also observed in the epithelium after day 6 (Fig. 2D,D′). The expression of SOX1/SOX2/PAX6 became more apparent in the epithelial structure at day 9 (Fig. 2B″,C″,D″). By contrast, under SMN protocol condition, no continuous epithelial structure was formed from days 3 to 9. Instead a SOX2^+^/PAX6^+^ rosette-like structure was formed in the aggregates (Fig. S1G-H″). The continuous epithelium under 3-DiSC condition widely expressed N-cadherin on day 9, and the expression pattern was dense in the outer side of the epithelium (Fig. 2G). The expression of apical marker aPKC was also observed mainly in the same area, indicating the continuous epithelial structure formed an apical surface on its outside from the early stage of differentiation (Fig. 2G′). On day 9, the expression of Lmx1a was observed on the small protrusions (Fig. 2H). These observations confirmed the generation of a neuroepithelium structure up to day 6 and the emergence of Lmx1a^+^ roof plate-like small protrusions at day 9 under the 3-DiSC condition.

Because BMP inhibitors, which are known to promote neural differentiation, were removed under the 3-DiSC condition, we also evaluated other lineage cells. Regarding neural crest lineage, a small number of SOX10^+^ cells were generated, but they were confined to the inner part of the aggregates (Fig. 2E-E″). Concerning mesodermal lineage, brachyury^+^ cells could not be detected after day 3 (Fig. 2F-F″). These data suggested our 3-DiSC condition mainly induced neural lineage from the early stage of differentiation.

In the developing embryonic dorsal spinal cord, roof plate plays an important role as a dorsal organizing center that produces BMPs and Wnts, which are responsible for the patterning of the dorsal spinal cord (Chizhikov and Millen, 2004b; Lee et al., 2000). We speculated the involvement of roof plate-like morphogen-generating structures in the generation of the patterned dorsal spinal cord tissue under our 3-DiSC condition, and assessed the expression pattern of BMP and Wnt signaling-related markers around the roof-plate-like protrusions. On day 9, MSX1 and MSX2, both of which are downstream targets of BMPs, were expressed in a region beyond the Lmx1^+^ region (Fig. 2H′). Other downstream targets of BMPs, such as phospho-Smad 1/5, were also observed in the protrusion (Fig. 2I,I′), and the expression of Wnt1 was detected around the Lmx1a^+^ regions (Fig. 2J,J′). These observations are consistent with the idea that Lmx1a^+^ protrusions play a role as an organizing center by producing inductive signals such as BMP and Wnt (Fig. 2K). Based on the above, we concluded that our 3-DiSC condition could efficiently induce continuous neuroepithelium for the first 6-9 days with self-forming roof plate-like protrusions, which have the potential to produce inductive signals.

### BMP4 treatment dorsalized the identity of spinal cord-like tissues

**Fig. 3.**
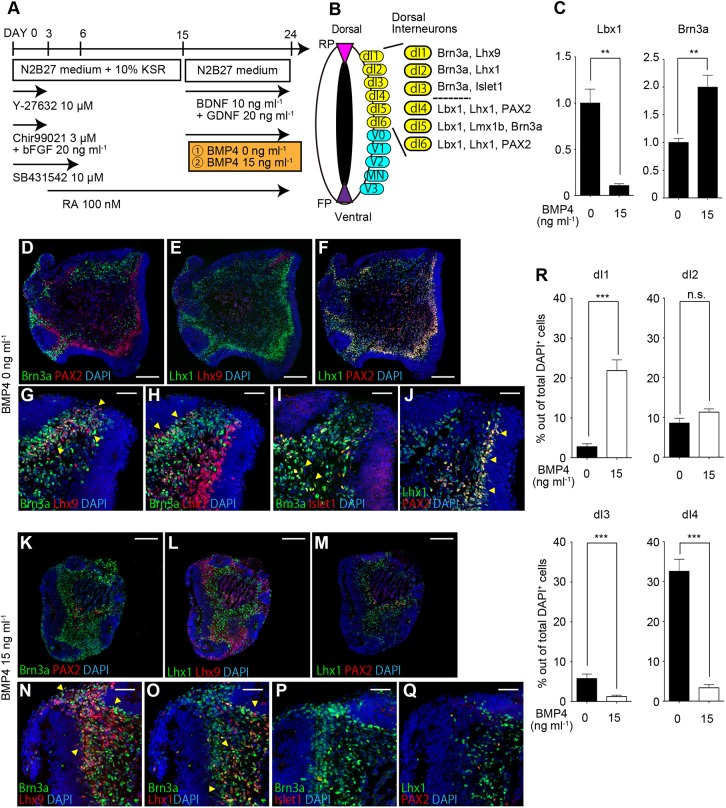
**BMP4 treatment dorsalized the identity of spinal cord-like tissues.** (A) Schematic of the examined conditions. (B) Schematic showing the marker expression of each dorsal interneuron subclass. (C) qPCR analysis on day 24 showing the relative expression of dorsal interneuron markers under BMP4-treated condition compared with non-treated condition (**P<0.01, *n*=7 total RNA samples from three independent culture experiments, unpaired *t*-test with Welch's correction, two-tailed). Data are mean±s.e.m. (D-J) Immunostaining under BMP 0 ng ml^−1^ condition on day 24. (D) Brn3a^+^ and PAX2^+^ populations exhibited separate distributions. (E) An Lhx9^+^ population was observed on the tip of the aggregates. (F) The PAX2^+^ population co-expressed Lhx1. (G-J) Lhx9^+^/Brn3a^+^, Lhx1^+^/Brn3a^+^, Islet1^+^/Brn3a^+^ and PAX2^+^/Lhx1^+^ populations were suggested to be dI1, dI2, dI3, and dI4 or dl6, respectively. (K-Q) Immunostaining under BMP 15 ng ml^−1^ condition on day 24. (K) A Brn3a^+^ population was mainly generated. (L) Lhx9^+^ and Lhx1^+^ populations exhibited separate distributions. (M) PAX2^+^/Lhx1^+^ cells were rarely observed. (N,O) Lhx9^+^/Brn3a^+^ (dI1) and Lhx1^+^/Brn3a^+^ (dI2) populations were mainly generated. (P,Q) Islet1^+^/Brn3a^+^(dI3) and PAX2^+^/Lhx1^+^ (dI4 or dl6) populations were rarely observed. (R) Percentages of each population among total DAPI^+^ cells. Data are mean±s.e.m. (***P<0.001, n.s., not significant; *n*=9 aggregates from three independent experiments). Scale bars: 200 μm in D-F,K-M; 50 μm in G-J,N-Q.

Next, we tried to further dorsalize the induced spinal cord-like tissues. In the developing spinal cord, dorsal interneurons (dIs) are generated toward the outside of the progenitor zone. Dorsal interneurons are divided into six discrete subclasses (dI1-6) and can be distinguished by the combination of expressed transcription factors (Fig. 3B, Fig. S3A-F) (Müller et al., 2002; Gross et al., 2002). As BMP4 is required for the generation of the dorsal-most subclass, we compared BMP4-treated conditions and non-treated conditions (Fig. 3A) (Le Dreau et al., 2012). We first examined the expression of dorsal interneuron markers using qPCR. Compared with non-treated conditions, the expression of Lbx1 (a marker for dI4-6) was significantly repressed upon BMP4 treatment, whereas the expression of Brn3a (a marker for dI1-3 and dl5) was upregulated (Fig. 3C). These results suggested that BMP4 treatment dorsalized the character of the generated interneurons.

Next, we focused in more detail on the detection and characterization of each dorsal interneuron population by IHC. Under non-treated conditions, both Brn3a^+^ and PAX2^+^ populations were observed in different regions (Fig. 3D). In the Brn3a^+^ population cells, Lhx9^+^, Lhx1^+^ and Islet1^+^ cells were identified, suggesting them to be dI1, dl2 and dl3, respectively (Fig. 3E,G-I). PAX2^+^ cells co-expressed Lhx1, suggesting that they were dI4 or dl6 (Fig. 3F,J). These findings indicated that four types of dorsal interneuron marker-positive cells were detected under non-treated conditions. Under BMP4-treated conditions, the proportion of PAX2^+^ cells was remarkably decreased, whereas the Brn3a^+^ population increased (Fig. 3K). Among Brn3a^+^ cells, Lhx9^+^ and Lhx1^+^ cells were distributed separately (Fig. 3L,N,O). Only a few Brn3a^+^/Islet1^+^ cells and PAX2^+^/Lhx1^+^ cells were detected (Fig. 3M,P,Q). Compared with non-treated conditions, the percentage of the dI1 marker-positive cell population was significantly increased, that of dI2 was unchanged, and those of dI3 and dI4 were significantly decreased (Fig. 3R). This BMP treatment did not affect SOX2 expression and did not promote SOX10^+^ neural crest cell generation (Fig. S4A,B). To investigate whether other BMP subtypes could dorsalize the induced spinal cord-like tissues, we tested BMP7 treatment. Under BMP7-treated conditions, dI4/6 marker-positive (Lhx1^+^/PAX2^+^) cells were decreased and dI1 marker-positive (Brn3a^+^/Lhx9^+^) cells were increased compared with the non-treated condition, indicating BMP7 treatment could also dorsalize the tissues (Fig. S4C-C″″,D-D″″). These results indicate that BMP treatment dorsalized the induced spinal cord-like tissues, mainly giving rise to dI1 and dI2 marker-positive cells.

Next, we examined the interplay between retinoic acid (RA) and BMPs. As RA activates the expression of PAX6, while BMP signaling represses the expression of PAX6 and induces dorsal-most progenitor domains (Novitch et al., 2003; Timmer et al., 2002), we hypothesized that the elimination of RA would enhance the effect of BMP signaling and dorsalize the generated cellular character. We tested the effect of RA removal between days 15 and 24 on the two BMP4 groups. Concerning the non-treated group, RA removal decreased dI4/6 marker-positive (PAX2^+^/Lhx1^+^) cells, but increased dI1 marker-positive (Brn3a^+^/Lhx9^+^) cells (Fig. S5A-A′″,B-B′″). Concerning the BMP4-treated group, RA removal increased dI1 marker-positive (Brn3a^+^/Lhx9^+^) cells, but decreased dI2 marker-positive (Brn3a^+^/PAX2^+^) cells (Fig. S5C-C′″,D-D′″). These data suggest that the removal of RA also leads to the dorsalization of generated cellular character (Fig. S5E). Collectively, these results demonstrate that our 3-DiSC condition can induce spinal cord-like tissue with four different types of dorsal interneurons, and that the cellular character of the generated cell populations can be changed and dorsalized by BMP4 treatment or RA removal.

### Spinal cord-like tissues were ventralized by activating Shh signaling in a dose-dependent manner

**Fig. 4.**
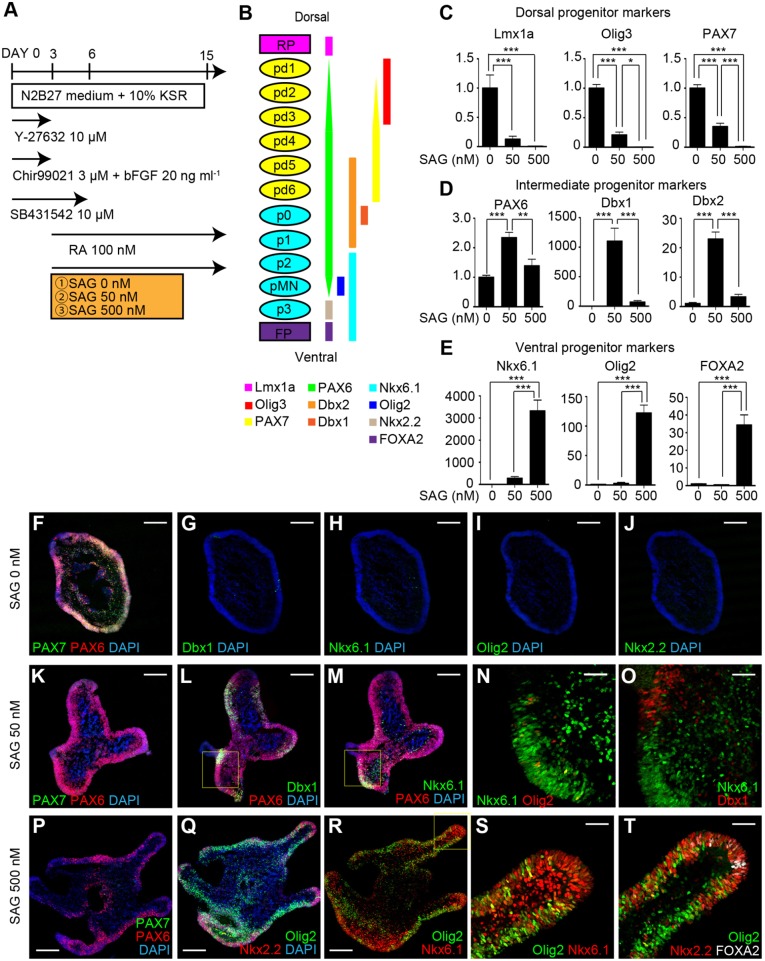
**Spinal cord-like tissues were ventralized by activating Shh signaling in a dose-dependent manner.** (A) Schematic of the examined conditions. (B) Schematic showing the expression pattern of progenitor domain markers in the developing spinal cord. (C-E) qPCR analysis on day 15 showing the relative expression of dorsal progenitor markers (C), intermediate progenitor markers (D) and ventral progenitor markers (E). Fold changes compared with SAG 0 nM (*P<0.05, **P<0.01, ***P<0.001, *n*=7 total RNA samples from three independent culture experiments, one-way analysis of variance followed by post-hoc Tukey's test). Data are mean±s.e.m. (F-J) Immunostaining under SAG 0 nM on day 15. PAX7 was widely expressed, whereas no ventral markers such as Dbx1 (G), Nkx6.1 (H), Olig2 (I) or Nkx2.2 (J) were detected. (K-O) Immunostaining under SAG 50 nM. (K) The continuous epithelium expressed PAX6 but not PAX7. (L,M) Dbx1^+^ and Nkx6.1^+^ domains were induced. (N) Olig2 was scarcely expressed in Nkx6.1^+^ domains. (O) Dbx1^+^ and Nkx6.1^+^ domains did not adjoin each other. (P-T) Immunostaining under SAG 500 nM. (P) The continuous epithelium partially expressed PAX6 but not PAX7. (Q) Olig2^+^ and Nkx2.2^+^ domains were mainly induced. (R,S) Nkx6.1 was widely expressed. (T) FOXA2^+^, Nkx2.2^+^ and Olig2^+^ domains aligned similarly to how they aligned in *in vivo* developing ventral spinal cord. Scale bars: 200 μm in F-M,P-R; 50 μm in N,O,S,T.

Next, we tried to ventralize the hPSC-derived spinal cord-like tissues. Because a Shh gradient contributes to the patterning of ventral progenitor domains in the developing spinal cord, we examined the effect of SAG treatment on *in vitro*-derived 3D human spinal cord-like tissues to mimic the activation and presence of Shh signaling (Fig. 4A) (Ribes and Briscoe, 2009). We tested three conditions: SAG 0 nM (control), SAG 50 nM (moderate) and SAG 500 nM (strong). We initially assessed the gene expression of progenitor domain markers using qPCR (Fig. 4B) (Lu et al., 2015). Under SAG 50 nM and SAG 500 nM, the dorsal domain markers Lmx1a, Olig3 and PAX7 were downregulated (Fig. 4C). Regarding intermediate domain markers, the expression of Dbx1 and Dbx2 was significantly increased under SAG 50 nM, but not under SAG 500 nM (Fig. 4D). On the other hand, the expression of ventral domain markers such as Nkx6.1, Olig2 and FOXA2 was unperturbed by SAG 50 nM, but increased significantly under SAG 500 nM (Fig. 4E). These results suggested that SAG treatment could ventralize the progenitor domain identity in a dose-dependent manner.

To evaluate the structure, we further analyzed the expression patterns of progenitor domain markers in the aggregates by IHC. Continuous epithelial structures were formed under all conditions (Fig. S6A,E,I). Under SAG 0 nM, the expression of both PAX6 and PAX7 was observed in the continuous epithelium, but no expression of intermediate or ventral progenitor markers, such as Dbx1, Dbx2, Olig2 or Nkx2.2, was observed, which confirmed the generation of dorsal progenitor domains (Fig. 4F-J, Fig. S6A-D).

Under SAG 50 nM, PAX7 was not expressed, but PAX6 expression was maintained, suggesting the induction of ventral progenitor domains (Fig. 4K, Fig. S6E-H). In the PAX6^+^ continuous epithelium, Dbx1^+^ and Nkx6.1^+^ cells were observed to cover distinct regions within the epithelium (Fig. 4L,M). Dbx1^+^ regions were suggested to be the p0 domain. In the Nkx6.1^+^ regions, the expression of Olig2 was scarcely observed, suggesting Nkx6.1^+^ regions to correspond to the p2 domain (Fig. 4N). Furthermore, the p0 domain (Dbx1^+^) and p2 domain (Nkx6.1^+^/Olig2^−^) regions were not adjoined (Fig. 4O). Evx1^+^ (marker for V0) cells and Chx10^+^ (marker for V2a) cells were generated on day 24 (Fig. S6O,P). Together, these findings suggested the formation of an intermediate (ventral-dorsal) spinal cord-like structure under SAG 50 nM.

Under SAG 500 nM, the expression of PAX7 was again absent, but the expression of PAX6 was partially maintained (Fig. 4P, Fig. S6I,J). In the continuous epithelium, Olig2^+^ regions and Nkx2.2^+^ regions could be identified, suggesting the generation of the pMN domain and p3 domain, respectively (Fig. 4Q). Nkx6.1 was expressed widely in the same areas as Olig2^+^ (marker for pMN) and Nkx2.2^+^ regions (marker for p3), which is consistent with the expression patterns observed *in vivo* (Fig. 4R,S). The expression of FOXA2 (a marker for floor plate) was also observed in the aggregates (Fig. 4T, Fig. S6K,L). Some FOXA2^+^ cells co-expressed Arx, suggesting the generation of definitive floor plate (Fig. S6M) (Cho et al., 2014; Mansour et al., 2014). Notably, pMN (Olig2^+^), p3 (Nkx2.2^+^) and FP (FOXA2^+^) domains were aligned in a manner similar to the pattern observed during development of the ventral spinal cord *in vivo* (Fig. 4T) (Ribes et al., 2010). Hb9^+^, Islet1^+^ and Hb9^+^Islet1^+^ cells were generated on day 24, and some co-expressed Foxp1, suggesting the generation of spinal motor neurons, including lateral column type (Fig. S6Q-S). These findings suggested the formation of ventral spinal cord-like structures under SAG 500 nM. To summarize, these results demonstrated that SAG treatment ventralized the progenitor domain identity in a dose-dependent manner, making it possible to induce several distinct domains within a continuous neuroepithelial structure and to recapitulate the patterning of the developing intermediate and ventral spinal cord.

Furthermore, we evaluated the positional identity of the aggregates along their rostro-caudal (R-C) axis under SAG 500 nM. The positional identities are determined by the expression patterns of genes in the HOX loci (Fig. S7A) (Philippidou and Dasen, 2013). On day 15, qPCR analysis clearly detected the expression of HOXC5 and HOXC6 but not of HOXC8, HOXC9 or HOXC10, suggesting the induced ventral spinal cord tissues corresponded to rostral cervical level spinal cord (Fig. S7C). Because FGFs play key roles in inducing and regulating progressive HOX activation, we tested the effect of prolonged FGF treatment (6 days; long-FGF condition) and compared the expression pattern of HOX genes with the control condition (Fig. S7B) (Mazzoni et al., 2013; Lippmann et al., 2015). Under the long-FGF condition, the expression of HOXC5 was not significantly changed, but the expression of HOXC9 was upregulated strongly (Fig. S7D). The expression of HOXC10 was still not evident. These results suggested the induction of thoracic levels under long-FGF. To summarize, rostral cervical level was induced under SAG 500 nM and the positional identity could be caudalized up to the thoracic level by prolonging FGF treatment.

### Generation of two distinct V2 interneuron subtypes

**Fig. 5.**
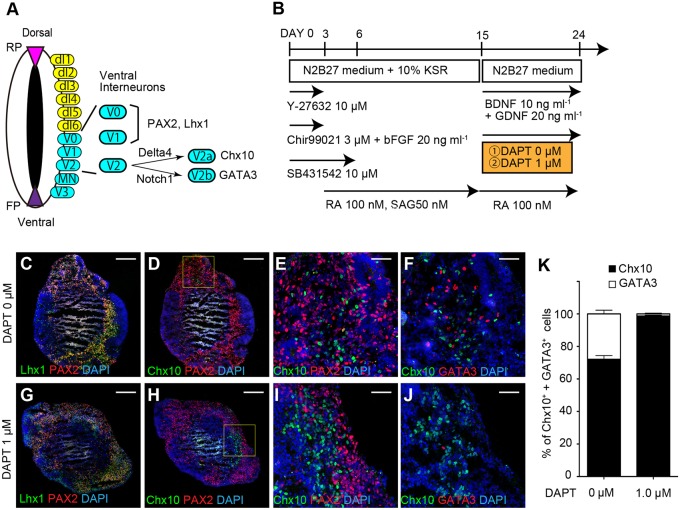
**Generation of two distinct V2a interneuron subclasses.** (A) Schematic showing the marker expression of V0-2 interneurons. (B) Schematic of the examined conditions. (C-F) Immunostaining under DAPT 0 μM on day 24. (C) An Lhx1^+^/PAX2^+^ population was suggested to be V0 or V1. (D-F) Both Chx10^+^ (V2a marker) cells and GATA3^+^ (V2b marker) cells were detected. (G-J) Immunostaining under DAPT 1 μM. Although Chx10^+^ (V2a marker) cells were observed, only a few GATA3^+^ (V2b marker) cells were detected. (K) Percentages of each population (*n*=9 aggregates from three independent experiments). Data are mean±s.e.m. Scale bars: 200 μm in C,D,G,H; 50 μm in E,F,I,J.

In the developing ventral spinal cord, the p2 domain yields several types of interneurons. Among them, the balance of excitatory V2a interneurons and inhibitory V2b interneurons is controlled by Notch signaling (Fig. 5A) (Joshi et al., 2009). The proportion of V2a interneurons is increased upon Notch inhibition. On the other hand, the specification of V2b interneurons requires Notch signaling. Because we identified p2 domains in the aggregates under SAG 50 nM, we examined the spinal cord-like 3D tissues on day 24 for the expression of V2a and V2b interneuron markers under the SAG 50 nM condition. IHC showed that the majority of cells expressed Lhx1 and PAX2, suggesting the presence of V0 or V1 interneurons (Fig. 5C). In addition, both Chx10^+^ (V2a interneuron marker) cells and GATA3^+^ (V2b interneuron marker) cells were observed in the analyzed 3D structures (Fig. 5D-F). These observations indicated that V0, V1, V2a and V2b marker-positive cells could be detected in 3D structures cultured under SAG 50 nM. In order to assess the effect of Notch signaling, we tested DAPT (Notch inhibitor) treatment and evaluated the composition of the V2 population (Fig. 5B). IHC showed a substantial number of Chx10^+^ cells, whereas GATA3^+^ cells were scarcely detected (Fig. 5G-J). Inhibition of Notch signaling increased the percentage of the Chx10^+^ population among total V2 interneuron marker-positive cells from 72.1% to 98.7% (Fig. 5K). These findings indicate that the division of V2a and V2b populations via Notch signaling could be recapitulated via our *in vitro* 3D induction system.

### Dissociated neurons show same expression patterns as somatosensory neurons and spinal motor neurons

**Fig. 6.**
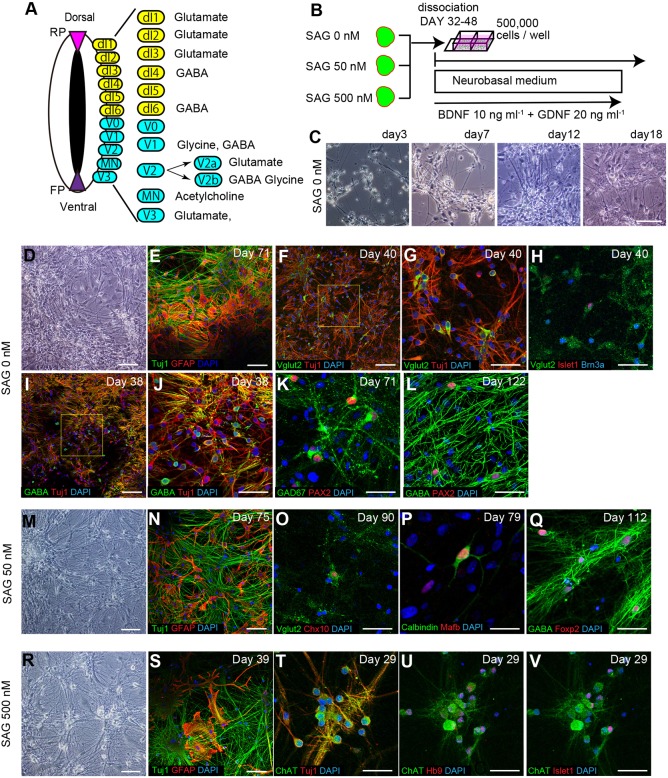
**Dissociated neurons showed expression patterns of somatosensory neurons and spinal motor neurons.** (A) Schematic showing the expression pattern of neurotransmitters. (B) Schematic of the protocol for dissociation culture. (C) Phase-contrast images in the early days after dissociation showing neurite extension and the emergence of flat-shaped cells. (D) Phase-contrast image under SAG 0 nM. (E) Immunostaining revealed the presence of both Tuj1^+^ neurons and GFAP^+^ glial cells. (F,G) Vglut2^+^/Tuj1^+^ glutamatergic neurons were detected. (H) Vglut2^+^ neurons expressing Brn3a or Islet1 were suggested to be somatosensory relay neurons derived from dI1-3. (I,J) GABA^+^/Tuj1^+^ neurons were detected. (K,L) GAD67^+^ and GABA^+^ neurons expressing PAX2 were suggested to be dI4-derived somatosensory associative neurons or dI6 interneurons. (M) Phase-contrast image under SAG 50 nM. (N) Both Tuj1^+^ neurons and GFAP^+^ glial cells were generated. (O) Vglut2^+^/Chx10^+^ neurons were suggested to be V2a interneurons. (P,Q) Calbindin^+^/MafB^+^ neurons and GABA^+^/Foxp2^+^ neurons were suggested to be V1 Renshaw interneurons and non-Renshaw V1 interneurons, respectively. (R) Phase-contrast image under SAG 500 nM. (S) Both Tuj1^+^ neurons and GFAP^+^ glial cells were generated. (T) ChAT^+^/Tuj1^+^ cholinergic neurons were detected. (U,V) ChAT^+^ neurons expressed Islet1 or Hb9, or both, suggesting the generation of spinal motor neurons. Scale bars: 100 μm in C-F,I,M,N,R,S; 50 μm in G,H,J-L,O,Q,T-V; 25 μm in P.

Last, we examined whether hPSC-derived dorsal or ventral spinal cord-like tissues could generate subtypes of spinal interneurons or spinal motor neurons. Each subclass of spinal neurons expresses a defined set of neurotransmitters (Fig. 6A) (Lu et al., 2015). Cholinergic spinal motor neurons express choline acetyltransferase (ChAT); dI1-3-derived somatosensory relay neurons and V2a interneurons are glutamatergic neurons that express Vglut2; and dI4-derived somatosensory associative neurons and dI6 interneurons are GABAergic neurons that express GAD67.

Because it is difficult to evaluate the character of a single neuron in 3D aggregates, we performed dissociation cultures, which enabled us to evaluate the presence of distinct neurotransmitters. The spinal cord-like aggregates generated under the different SAG conditions were dissociated around day 40 and plated on poly-D-lysine-laminin-fibronectin-coated plates (Fig. 6B). After dissociation, the plated cells started to elongate axons and form networks (Fig. 6C). On day 18 after the dissociation, flattened glial shaped cells also emerged (Fig. 6C, day 18).

Under all conditions, IHC confirmed the generation of a Tuj1^+^ neuronal population and a GFAP^+^ glial population (Fig. 6D,E,M,N,R,S). Under SAG 0 nM, among Tuj1^+^ neurons, the primary populations were Vglut2^+^ neurons and GABA^+^ neurons (Fig. 6F,G,I,J). Vglut2^+^ neurons expressed Brn3a or Islet1, suggesting that they were somatosensory relay neurons derived from dI1-3 (Fig. 6H). GAD67^+^ and GABA^+^ neurons expressed PAX2, suggesting they were dI4-derived somatosensory associative neurons or dI6 interneurons (Fig. 6K,L). Under SAG 50 nM, IHC also showed Vglut2^+^ neurons expressing Chx10, calbindin^+^ neurons expressing MafB and GABA^+^ neurons expressing Foxp2, suggesting that they were glutamatergic V2a interneurons, Renshaw interneurons and non-Renshaw V1 interneurons, respectively (Fig. 6O-Q) (Stam et al., 2012). Under SAG 500 nM, the main population consisted of ChAT^+^ neurons (Fig. 6T). These neurons expressed Islet1 or Hb9, or both, suggesting the generation of spinal motor neurons (Fig. 6U,V). To summarize, spinal cord-like tissues induced from hPSCs under defined conditions enabled the *in vitro* generation of several types of human spinal neurons, including somatosensory relay neurons, somatosensory associative neurons and spinal motor neurons.

## DISCUSSION

In this report, we demonstrated the 3D induction of spinal cord-like tissues from human PSCs. By modifying a previously described protocol for *in vitro* spinal motor neuron induction (SMN protocol), we successfully induced dorsal spinal cord-like tissues and generated four types of dorsal interneuron marker-positive cell populations. The character of these tissues could be dorsalized by BMP4 treatment. On the other hand, activation of Shh signaling led to the derivation of intermediate and ventral spinal cord-like tissues in a dose-dependent manner. These *in vitro*-derived spinal cord-like tissues were confirmed to give rise to several subclasses of spinal neurons (Fig. S8).

Most notably, our method enabled the simultaneous induction of several domains and maintained the induced patterns in a 3D structure. By omitting several factors, which usually restrict the direction of differentiation, we could induce a wider region of the dorsal spinal cord, including the Lmx1a^+^ roof plate region. Furthermore, these dorsal spinal cord-like tissues could generate four types of dorsal interneurons, which each exhibited distinct distribution patterns. Although some previous reports have demonstrated the generation of dorsal interneurons, none has provided a detailed evaluation of the neuronal subclasses in 3D structure (Meinhardt et al., 2014; Maury et al., 2015; Gupta et al., 2018). In contrast, our culture condition enabled the stepwise efficient induction of dorsal interneurons and their respective subclasses from hPSCs in 3D. Our 3-DiSC condition is expected to increase understanding of the development of the human somatosensory system, which is mainly organized by dorsal interneurons.

Besides the generation of several subclasses of spinal neurons, the 3D induction of spinal cord tissues enabled the simultaneous generation of roof plate-like organizing centers along with the neuroepithelium. The generation of the roof plate-like structure might be a key factor for the patterned formation of dorsal progenitor domains. We suggest a possible mechanism as follows. First, a small region of the epithelial structure begins to express Lmx1a. The Lmx1a^+^ domains then go on to express inductive factors, including BMPs and Wnts, allowing them to function as a dorsalizing organizing center. Finally, these inductive factors affected the surrounding neuroepithelium, making it possible to induce several dorsal domains with pattern formation, which is consistent with *in vivo* tissue. Previous *in vivo* studies have shown that Lmx1a is sufficient for roof plate induction and several components of roof plate signaling, including BMP4, GDF7 and Wnt1 (Chizhikov and Millen, 2004a). These signals are all essential for dorsal spinal cord patterning, but their roles depend on the factors and their downstream targets, such as MSX1-3 (Duval et al., 2014; Chizhikov and Millen, 2005; Liu et al., 2004). A future investigation of hPSC-derived spinal cord induction should focus on the mechanistic analysis, especially with regards to the factors that determine the self-organization of patterned spinal cord neuroepithelium.

Our 3-DiSC condition is also applicable to the induction of ventral spinal cord, including ventral interneurons. Because V0-V3 interneurons constitute the core elements of the local locomotor circuitry, they have been studied from a variety of viewpoints (Goulding, 2009; Benito-Gonzalez and Alvarez, 2012; Borowska et al., 2013; Bikoff et al., 2016). Some recent studies have described the efficient generation of V2a interneurons from mouse and human PSCs (Brown et al., 2014; Butts et al., 2017). In comparison with these studies, which focused on single populations, the advantage of our condition is that multiple ventral progenitor domains can be orderly induced in a single 3D structure. Because little is known about the mechanism regulating the distribution of interneuron and motor neuron subclasses within the developing spinal cord, our approach will provide novel insights on the development, patterning and overall organization of human ventral spinal cord tissue.

Our approach mimics the core aspects of human spinal cord development, but it also comes with important limitations. First, it is difficult to induce entire spinal cord tissues that contain all domains ranging from the dorsal side to ventral side. Because our culture system cannot provide concentration gradients of small molecules in the culture medium, it is difficult to induce two organizing centers with opposing roles at the same time. To overcome this problem, sophisticated microfluidic culture systems might be necessary. Second, induced spinal cord-like tissues showed inverted organization, with the neural progenitor cells on the outside of the structure where they formed the apical side and differentiated neural cells on the inside of the structure. A similar inversion was observed in other hPSC-derived *in vitro* neural tissues (Sakaguchi et al., 2015; Muguruma et al., 2015; Kuwahara et al., 2015). The mechanism regulating apico-basal polarity remains to be clarified, however, because these inverted structures were seen only in human PSC-derived tissues, differences in species or PSC state (e.g. primed versus naive) should be considered as candidate causes. Third, it is difficult to caudalize the positional identity of the induced spinal cord-like tissues toward lumbosacral levels. This result is consistent with a previous study showing that GDF11 is necessary to activate lumbosacral HOX genes (Lippmann et al., 2015). Modifications of the protocol to promote further caudalize positional identity is being considered. Fourth, we could not observe a clear formation of motor columns. One of the reasons might be the fact that the specification of motor neuron columns is related to not only dorso-ventral patterning but also complex cross-interactions with HOX effectors (Dasen et al., 2008; Philippidou et al., 2012; Mendelsohn et al., 2017). Our future challenge will be the recapitulation of motor neuron column formation in 3D and proper formation and regulation of the R-C axis.

The spinal cord is an essential tissue that connects the brain with other parts of the body. Although early developmental processes associated with spinal cord formation have been well studied using chick and mouse embryos, it still remains to be clarified how these complex network systems are correctly constructed during human development. Recently, tissue generation using hPSCs has been rigorously studied, including *in vitro* models of human central nervous system development (Muguruma et al., 2015; Kadoshima et al., 2013; Jo et al., 2016; Sakaguchi et al., 2015; Kuwahara et al., 2015). A combination of our 3D model of human spinal cord tissue development with cutting-edge technologies will likely contribute to a better understanding of the mechanisms and core principles underlying human neuronal network formation, especially of the ascending somatosensory and descending corticospinal system.

## MATERIALS AND METHODS

### Maintenance culture of human iPSCs

This study was approved by the ethics committees of Kyoto University, Kyoto, Japan. Human iPSCs (1039A1, 1231A3 and 1383D6) were maintained and cultured as previously described (Nishimura et al., 2016). Briefly, hiPSCs were maintained on LN511-E8-coated dishes with StemFit medium (Ajinomoto). For each passage, the cells were dissociated to single cells with Accumax (Innovative Cell Technologies) and replated at a density 1.3×10^4^ cells into each well of a six-well plate.

### Maintenance culture of hESCs

Human ESCs (KhES-1) were used in accordance with ‘The Guidelines for Derivation and Utilization of Human Embryonic Stem Cells’ of the Ministry of Education, Culture, Sports, Science and Technology of Japan after approval by the Institutional Review Board. hESCs were maintained and cultured as previously described (Sakaguchi et al., 2015). In brief, hESCs were maintained on a feeder layer of mouse embryonic fibroblasts inactivated by mitomycin C treatment in DMEM/F12 (Wako) supplemented with 20% knockout serum replacement (KSR, Invitrogen), 2 mM glutamine, 0.1 mM nonessential amino acids (Invitrogen), 5 ng ml^−1^ recombinant human basic fibroblast growth factor (bFGF) (Wako) and 0.1 mM 2-mercaptoethanol under 2% CO_2_. For passaging, hESC colonies were detached and recovered en bloc from the feeder layer by treating them with 0.25% (weight/vol) trypsin and 1 mg ml^−1^ collagenase IV in PBS containing 20% (vol/vol) KSR and 1 mM CaCl_2_ at 37°C for 7-8 min. The detached hESC clumps were broken into smaller pieces (several dozens of cells) by gentle pipetting. The passages were performed at a 1:4 to 1:6 split ratio every 4-5 days.

### Differentiation culture of human iPSCs

The differentiation culture for spinal motor neurons (SMN protocol) was performed as described previously (Maury et al., 2015). Human iPSCs were dissociated to single cells using Accumax and quickly reaggregated using low cell adhesion U-bottomed 96-well plates (Lipidure-Coat Plate A96-U, NOF corporation) in differentiation medium (9000 cells per well, 100 μl). The differentiation medium was N2B27 [DMEM/F-12 (Wako), neurobasal medium (Gibco) (1:1), 0.5% (vol/vol) N2 supplement (ThermoFisher) and 1% (vol/vol) B27 supplement without vitamin A (Invitrogen)] supplemented with 1 mM L-glutamine (L-Glu, Sigma-Aldrich), 0.1 mM 2-mercaptoethanol (2-ME, Wako) and 0.5 μM ascorbic acid (Towa). Half the medium was changed once every 3 days. Defining the day on which the differentiation culture was started as day 0, 10 μM Y-27632 (Wako), 20 ng ml^−1^ recombinant human basic fibroblast growth factor (bFGF, Wako) and 3 μM CHIR99021 (Stemgent) were added from day 0 to day 3, and 10 μM SB431542 (Tocris) and 0.2 μM LDN193189 (Stemgent) were added from day 0 to day 6. Retinoic acid (RA, 100 nM, Sigma-Aldrich) and 500 nM Smoothened agonist (SAG, Enzo) were added from day 3 to day 15.

For the induction of spinal cord tissues (3-DiSC condition), LDN193189 and SAG were removed from the SMN condition, and 10% (vol/vol) knockout serum replacement (KSR, Invitrogen) was added to the differentiation medium. Half the medium was changed once every 3 days. As for the differentiation culture of 1383D6, CHIR99021 treatment was reduced to 1.5 μM. As for the differentiation culture of Kh1, bFGF 20 ng ml^−1^ and 50 μM Y-27632 were added to the differentiation medium for the first 3 days. After day 3, the same differentiation protocol used for human iPSCs was applied.

On day 15, the floating aggregates were transferred from a 96-well plate to a 6 cm Petri dish (non-cell adhesive) and further cultured in suspension with N2B27 medium supplemented with 1 mM L-Glu, 0.1 mM 2-ME, 0.5 μM ascorbic acid, 10 ng ml^−1^ BDNF (Wako), 20 ng ml^−1^ GDNF (Wako) and 100 nM RA.

For dorsalization, 15 ng ml^−1^ BMP4 (R&D) was added from day 15 to day 24 (Fig. 2A). For ventralization, 50 nM or 500 nM SAG was added from day 3 to day 15 (Fig. 3A). For the inhibition of Notch signaling, 1 μM DAPT (Sigma-Aldrich) was added from day 15 to day 24 (Fig. 4B).

### Neuronal dissociation culture

Neuronal dissociation culture was performed as previously described (Sakaguchi et al., 2015). The aggregates were dissociated to single cells using the neural tissue dissociation kit (Sumitomo Bekelite, MB-X9901) on days 32-48 and plated onto poly-D-lysine/laminin/fibronectin-coated 2-well glass dishes at a density of 500,000 cells per well in neurobasal medium supplemented with 2% (vol/vol) B-27 supplement without vitamin A, 2 mM L-glutamine (Invitrogen), 10 ng ml^−1^ BDNF and 20 ng ml^−1^ GDNF. The medium was changed every 3 days.

### Histological study of mouse embryonic spinal cord

The experiments were performed according to the Guidelines for Animal Experiment of Kyoto University, the Guide for the Care and Use of Laboratory Animals of the Institute for Laboratory Animal Resources (Washington, DC, USA), and the Animal Research: Reporting *in vivo* Experiments (The ARRIVE guidelines). Histological studies of mouse embryonic tissues were performed as previously described (Samata et al., 2016). Pregnant mice (C57BL/6NCrSlc) were obtained from Shimizu Laboratory Supplies (Kyoto, Japan). E11.5 embryos were removed and fixed in 4% paraformaldehyde (4°C, 2 h). Following cryostat sectioning, the cervical spinal cord was assessed using IHC.

### Immunohistochemistry

IHC was performed as previously reported (Sakaguchi et al., 2015). In brief, aggregates were fixed in 4% paraformaldehyde, frozen in optimum cutting temperature (OCT) embedding medium and sectioned at 10 μm using a cryostat microtome. Serial sections of aggregates were attached onto slide glasses and incubated with primary antibodies (at 4°C overnight) following incubation with secondary antibodies conjugated with Alexa 488, 594 and 647 (at room temperature for 2 h). For the detection of GABA, 0.05% glutaraldehyde (Nacalai Tesque) was included in the fixative. All images were obtained using a confocal laser microscope (Olympus FV1000), except for wide-field images, which were obtained using a BioRevo fluorescence microscope (BZ-X710 Keyence). The primary antibodies used and their dilutions are listed in Table S1.

### Quantitative analysis of immunohistochemistry

For quantitative analysis of IHC, nine aggregates from three independent culture experiments were examined. Cell counting was performed using ImageJ. Regarding Fig. 3R, the number of Brn3a^+^/Lhx9^+^ (dI1), Brn3a^+^/Lhx1^+^ (dI2), Brn3a^+/^Islet1^+^ (dI3), PAX2^+^/Lhx2^+^ (dI4) and DAPI^+^ cells were counted in two high-magnification field images covering one side of each aggregate. The percentage of individual neuronal types among total DAPI^+^ cells were calculated. For Fig. 5K, Chx10^+^ (V2a) and GATA3^+^ (V2b) cells were counted in one representative high-magnification field image of each aggregate. The percentage of each individual neuronal type among total V2 interneurons (V2a+V2b) was calculated.

### Quantitative PCR

qPCR was performed as previously described (Nishimura et al., 2016). Each total RNA sample was extracted from 8-12 aggregates using RNeasy Plus Mini Kit (Qiagen), after which 1 μg of total RNA was used for reverse transcription by Super Script III First-Strand Synthesis System with Olig(dT)_20_ primer (Invitrogen). Amplification was performed with Power SYBR Green PCR Master Mix (ThermoFisher). qPCR was performed on a StepOne detection system (Applied Biosystems). The data were normalized to GAPDH expression. Primer sequences are listed in Table S2.

For quantitative analysis, three independent differentiation culture experiments were performed. One or more total RNA samples were prepared in each differentiation experiment. For Fig. 1H, six total RNA samples from three independent culture experiments were examined. For Fig. 3C, seven total RNA samples from three independent culture experiments were examined. For Fig. 4C-E, seven total RNA samples from three independent culture experiments were examined. For Fig. S7C,D, five total RNA samples from three independent culture experiments were examined.

### Bright field view time-lapse imaging of cell aggregation

The differentiation culture of hPSCs was performed as aforementioned with U-bottomed conical wells (Sumilon PrimeSurface plate; Sumitomo Bakelite) under 3-DiSC condition. The original imaging data were taken by IncuCyte S3 Spheroid Software Module (Essen Bioscience) every 2 h for 66 h from the start of differentiation. Images were processed by WCIF ImageJ software and Adobe Premiere Pro CC 2017.

### Statistical analysis

All data are shown as the mean±s.e.m. Statistical tests were performed with PRISM software (GraphPad version 5). For single comparisons, the statistical significance was tested by unpaired *t*-test or unpaired *t*-test with Welch's correction. For multiple comparisons, statistical significance was determined by one-way analysis of variance followed by Tukey's post hoc test.

## Supplementary Material

Supplementary information
