# Supplementary material for: Three-dimensional induction of dorsal, intermediate and ventral spinal cord tissues from human pluripotent stem cells
Source: Development. 2018 Jul 30;145(16):dev162214. doi: 10.1242/dev.162214 (PMC6124545; doi:10.1242/dev.162214)
Supplement: Supplementary information [file develop-145-162214-s1.pdf]

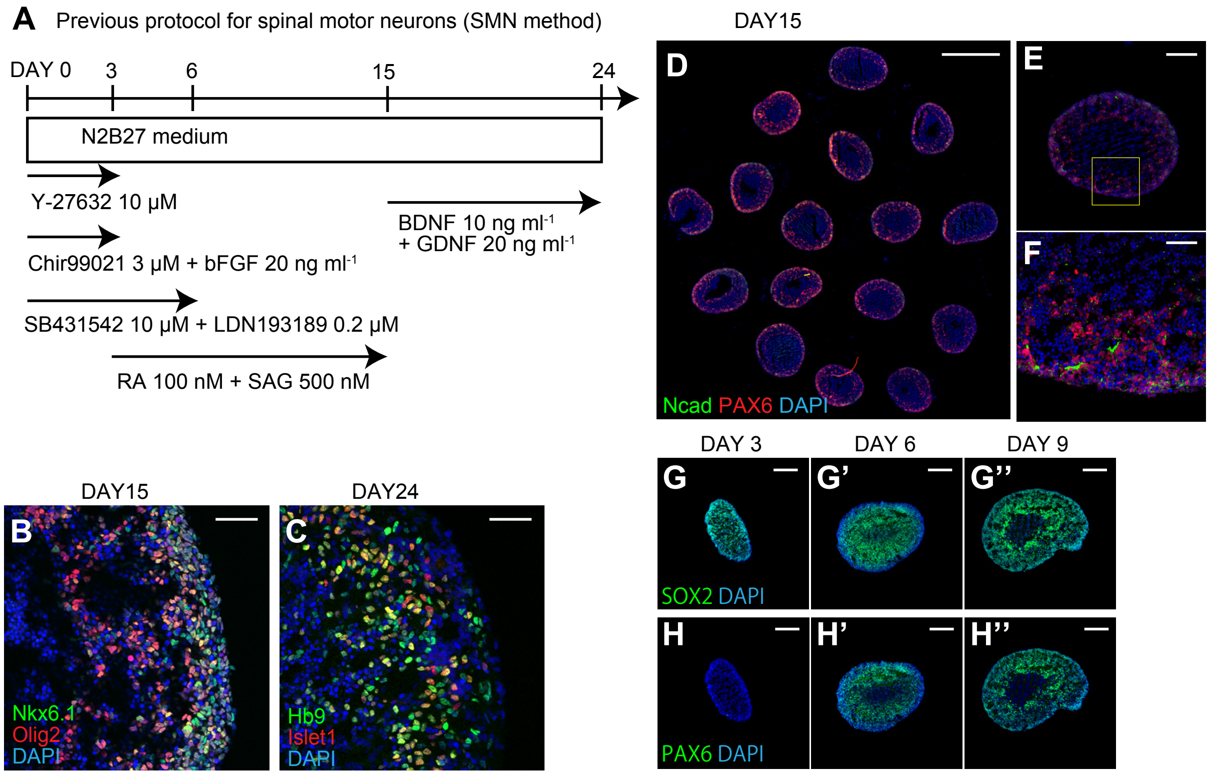

Fig. S1 Generation of spinal motor neurons using a previously described protocol. (A) Schematic of the differentiation protocol for spinal motor neurons (SMN protocol). (B) Nkx6.1$^+$/Olig2$^+$ spinal motor neuron progenitors were efficiently generated on day 15. (C) Hb9$^+$/Islet1$^+$ spinal motor neuron precursors were efficiently generated on day 24. (D-F) The expression of N-cadherin was observed in a discontinuous manner. (G-H'') SOX2$^+$/PAX6$^+$ rossette-like structure formation was observed up to day 9. Scale bars: 1000 μm (D); 200 μm (E, G-H''); 50 μm (B, C, F).

Development • Supplementary information

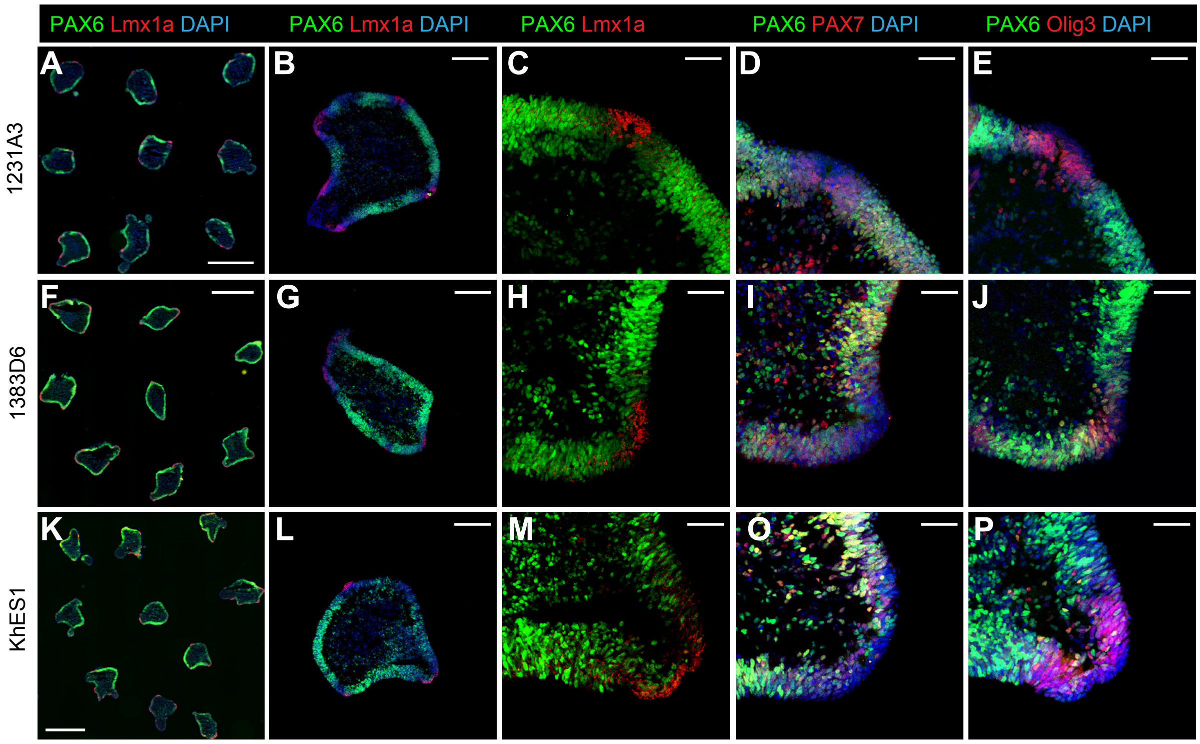

Fig. S2 Induction of dorsal spinal cord-like tissues from other hPSC lines. Dorsal spinal cord-like tissues could be induced using other human iPS cell lines (1231A1 (A-E), 1383D6 (F-J)) and a human ES cell line (KhES1 (K-P)). Continuous epithelium structure expressed PAX6 (A, E, I). Lmx1a^{+} domains were induced in PAX6^{-} regions (A-C, F-H, K-M). PAX6^{+} continuous epithelium co-expressed PAX7 (D, I, Q). Olig3^{+} domains were induced adjacent to Lmx1a^{+} regions (E, J, P). These structures were observed in 90.9% (1231A3), 83.3% (1383D6) and 57.5% (KhES1) of the analyzed aggregates in 3 independent experiments. Scale bars: 1000μm (A, F, K); 200 μm (B, G, L); 50 μm (C-E, H-J, M-P).

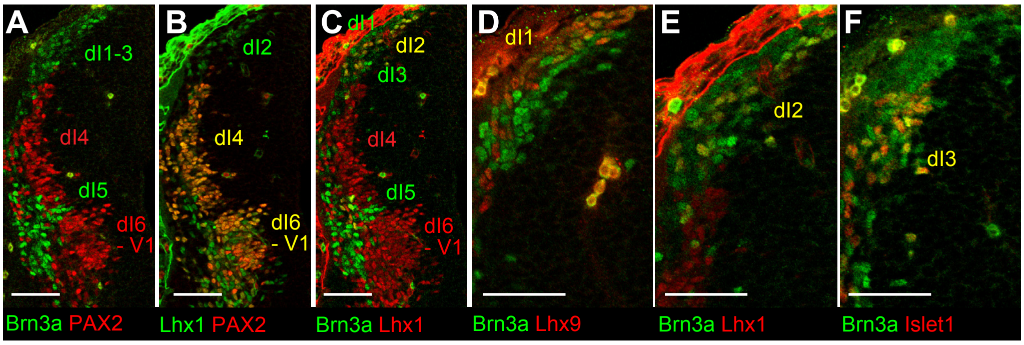

Fig. S3. Expression pattern of dorsal interneuron markers in mouse embryo E11.5. (A) Brn3a^{+} population and PAX2^{+} population exhibited separate distributions. (B) Lhx1^{+}/PAX2^{+} population was observed in the dorsal side and ventral side. (C) Lhx1^{+} cells in the dorsal side co-expressed Brn3a. (D-F) Among Brn3a^{+} cells, Lhx9^{+} cells (D), Lhx1^{+} cells (E), and Islet1^{+} cells (F) were dI1, dI2 and dI3, respectively. Scale bars:100 μm (A-C); 50 μm (D-F).

Development • Supplementary information

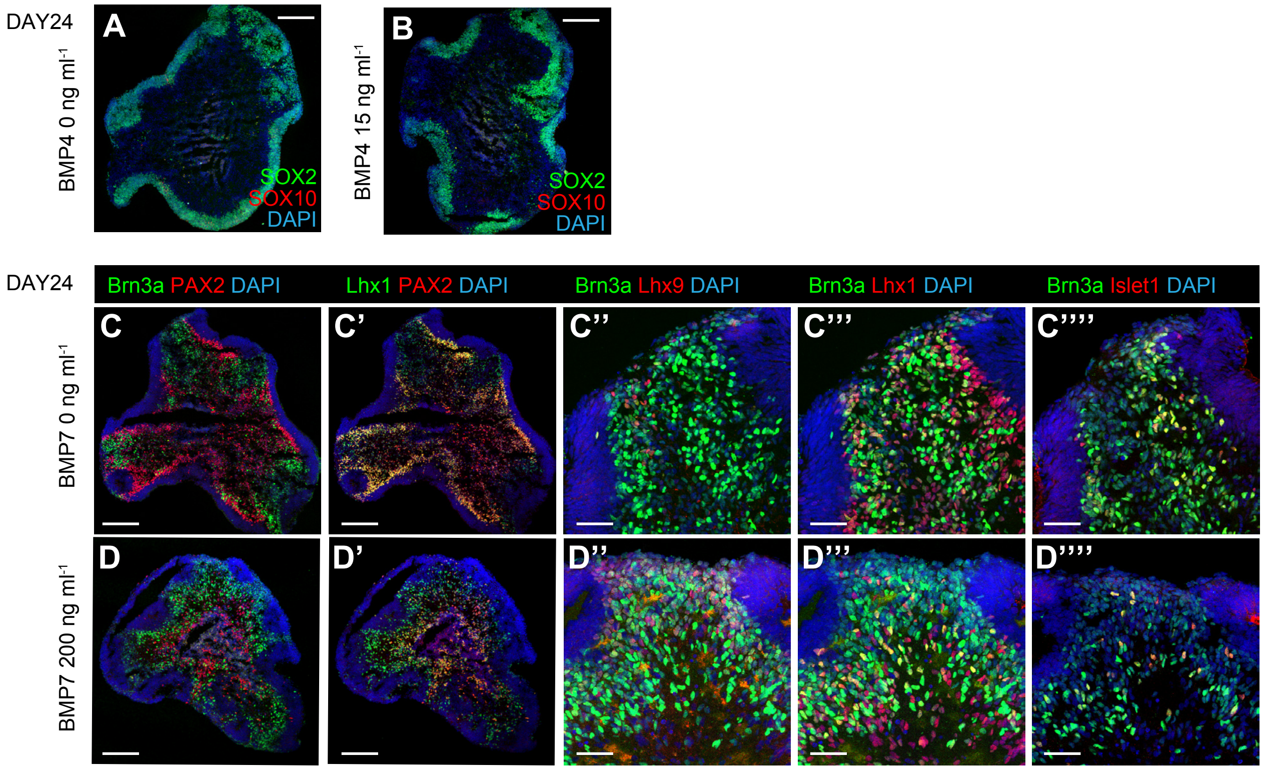

Fig. S4. BMP treatment dorsalized the identity of spinal cord-like tissues.
(A, B) Immunostaining for SOX2 and SOX10 on day 24 under BMP4 0 ng ml^{-1} (A) and BMP4 15 ng ml^{-1} (B). SOX2 was expressed in the epithelial structure, but $SOX10^{+}$ cells were rarely detected in (A). BMP4 treatment did not increase $SOX10^{+}$ cells. (C-C’’’’) Immunostaining under BMP7 0 ng ml^{-1} on day 24. (C) $Brn3a^{+}$ and $PAX2^{+}$ cell populations were detected. (C’) $PAX2^{+}$ populations co-expressed Lhx1, suggesting the generation of dI4 or dI6. (C’’-C’’’’) $Lhx9^{+}/Brn3a^{+}$, $Lhx1^{+}/Brn3a^{+}$, $Islet1^{+}/Brn3a^{+}$ populations were detected, suggesting the generation of dI1, dI2 and dI3 respectively. (D-D’’’’) Immunostaining under BMP7 200 ng ml^{-1} on day 24. (D) $Brn3a^{+}$ populations were mainly generated. (D’) $PAX2^{+}/Lhx1^{+}$ (dI4/6) cells were decreased compared with BMP7 0 ng ml^{-1}. (D’’-D’’’’) $Lhx9^{+}/Brn3a^{+}$ (dI1), $Lhx1^{+}/Brn3a^{+}$ (dI2) cells were mainly generated, while only a few number of $Islet1^{+}/Brn3a^{+}$ (dI3) cells were detected. Scale bars: 200 μm (A, B, C, C’, D, D’); 50 μm (C’’-C’’’’, D’’-D’’’’).

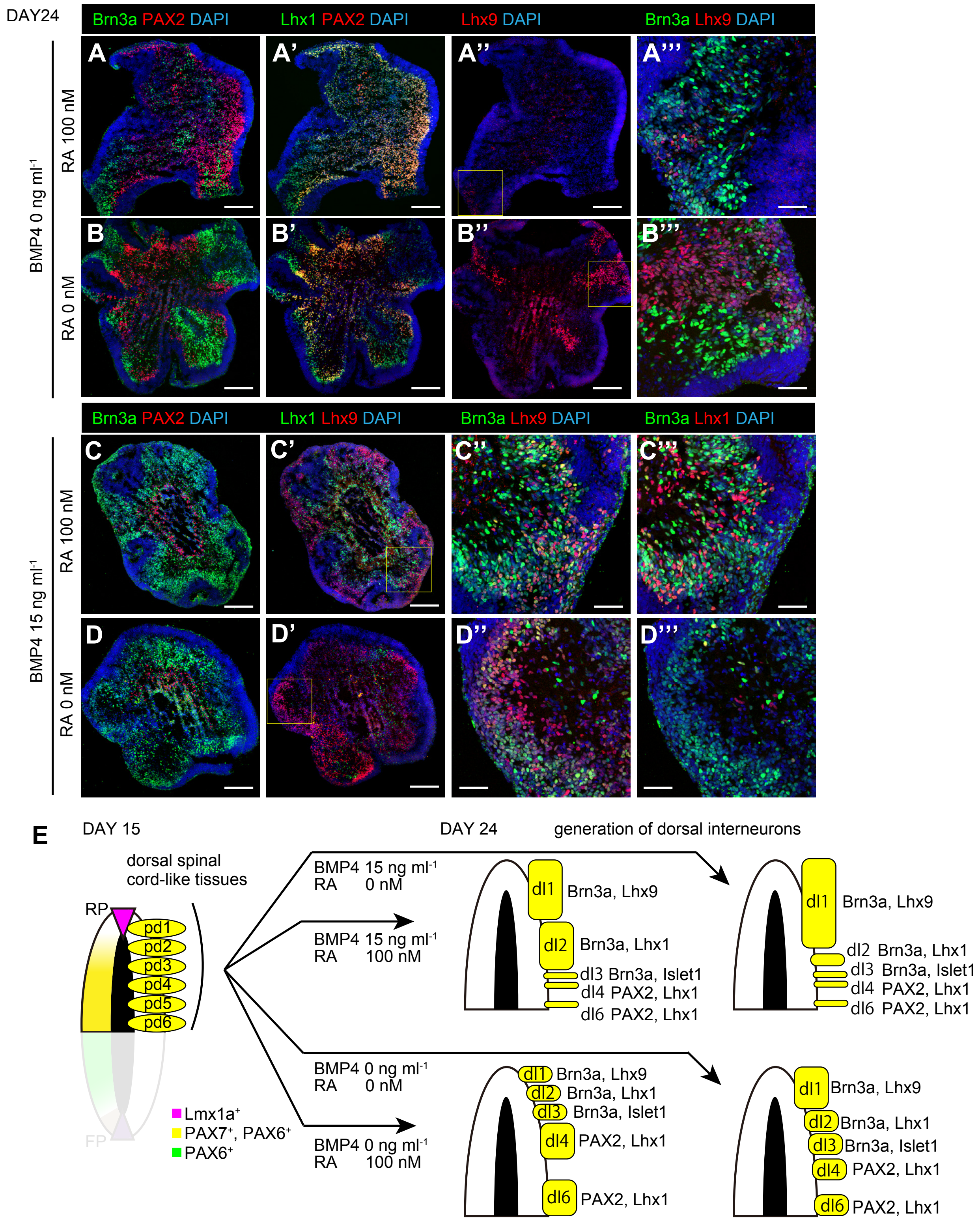
DAY24
Brn3a PAX2 DAPI
Lhx1 PAX2 DAPI
Lhx9 DAPI
Brn3a Lhx9 DAPI
BMP4 0 ng ml-1
RA 100 nM
RA 0 nM
A
A'
A''
A'''
B
B'
B''
B'''
Brn3a PAX2 DAPI
Lhx1 Lhx9 DAPI
Brn3a Lhx9 DAPI
Brn3a Lhx1 DAPI
BMP4 15 ng ml-1
RA 100 nM
RA 0 nM
C
C'
C''
C'''
D
D'
D''
D'''
E
DAY 15
dorsal spinal cord-like tissues
RP
pd1
pd2
pd3
pd4
pd5
pd6
FP
Lmx1a+
PAX7+, PAX6+
PAX6+
DAY 24
generation of dorsal interneurons
BMP4 15 ng ml-1 RA 0 nM
BMP4 15 ng ml-1 RA 100 nM
BMP4 0 ng ml-1 RA 0 nM
BMP4 0 ng ml-1 RA 100 nM
dI1 Brn3a, Lhx9
dI2 Brn3a, Lhx1
dI3 Brn3a, Islet1
dI4 PAX2, Lhx1
dI6 PAX2, Lhx1

Fig. S5. Examination of the interplay between BMPs and RA

(A, B) The role of RA between days 15 and 24 were examined under BMP4 0 ng ml^{-1} condition. (A-A''') Immunostaining under RA 100 nM on day 24. (A) Brn3a^{+} and PAX2^{+} cell populations were generated. (A') PAX2^{+} populations co-expressed Lhx1, suggesting the generation of dI4 or dI6. (A'',A''') Lhx9^{+}/Brn3a^{+} cells were detected, suggesting the generation of dI1. (B-B''') Immunostaining under RA 0 nM on day 24. (B, B') Compared with RA 100 nM, Brn3a^{+} cells were increased, while PAX2^{+}/Lhx1^{+} (dI4/6) cells were decreased. (B'', B''') Lhx9^{+}/Brn3a^{+} (dI1) cells were increased. (C, D) The role of RA between days 15 and 24 was examined under BMP4 15 ng ml^{-1} condition. (C-C''') Immunostaining under RA 100 nM on day 24. (C) Brn3a^{+} cells were mainly generated, while few PAX2^{+} cells were observed. (C'-C''') Lhx9^{+}/Brn3a^{+} (dI1) and Lhx1^{+}/Brn3a^{+} (dI2) cells were generated. (D-D''') Immunostaining under RA 0 nM on day 24. (D) Brn3a^{+} cells were mainly generated. (D'-D''') Lhx9^{+}/Brn3a^{+}(dI1) cells were mainly generated, while few Lhx1^{+}/Brn3a^{+} (dI2) cells were detected. (E) Schematic summary of the examined conditions. Scale bars: 200 µm (A-A'', B-B'', C, C', D, D'); 50 µm (A''', B''', C'',C''', D'', D''').

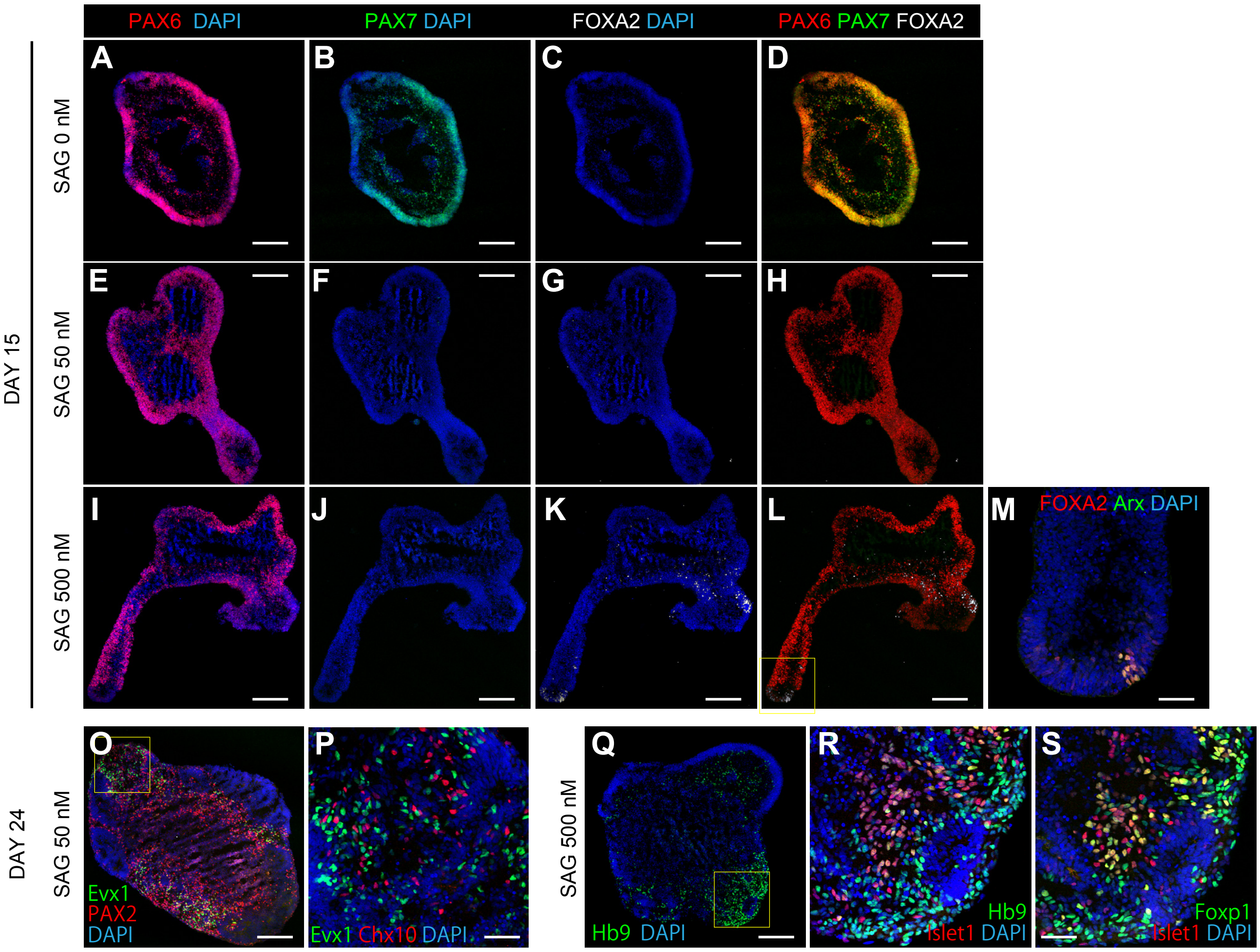

Fig. S6. Spinal cord-like tissues were ventralized by activating Shh signaling in a dose-dependent manner. (A-D) Under SAG 0 nM, continuous epithelium expressed both PAX6 (A) and PAX7 (B) but not FOXA2 (C, D). (E-H) Under SAG 50 nM, continuous epithelium expressed PAX6 (E), but not PAX7 (F) or FOXA2 (G, H). (I-L) Under SAG 500 nM, continuous epithelium partially expressed PAX6 (I), but not PAX7 (J). FOXA2 expression was also observed (K, L), suggesting the generation of the ventral-most part of the spinal cord. (M) Some of FOXA2$^+$ cells co-expressed Arx, suggesting the presence of definitive floor plate. (O) Evx1$^+$ cells were observed on day 24 under SAG 50 nM condition, suggesting the generation of V0. (P) Chx10$^+$ cells were detected, suggesting the generation of V2a. (Q, R) Hb9$^+$, Islet1$^+$ and Hb9$^+$/Islet1$^+$ cells were generated on day 24 under SAG 500 nM condition. (R) FOXP1$^+$ cells were observed, suggesting the generation of lateral motor column motor neurons. Scale bars: 200 µm (A-L, O, Q); 50 µm (M, P, R, S).

Development • Supplementary information

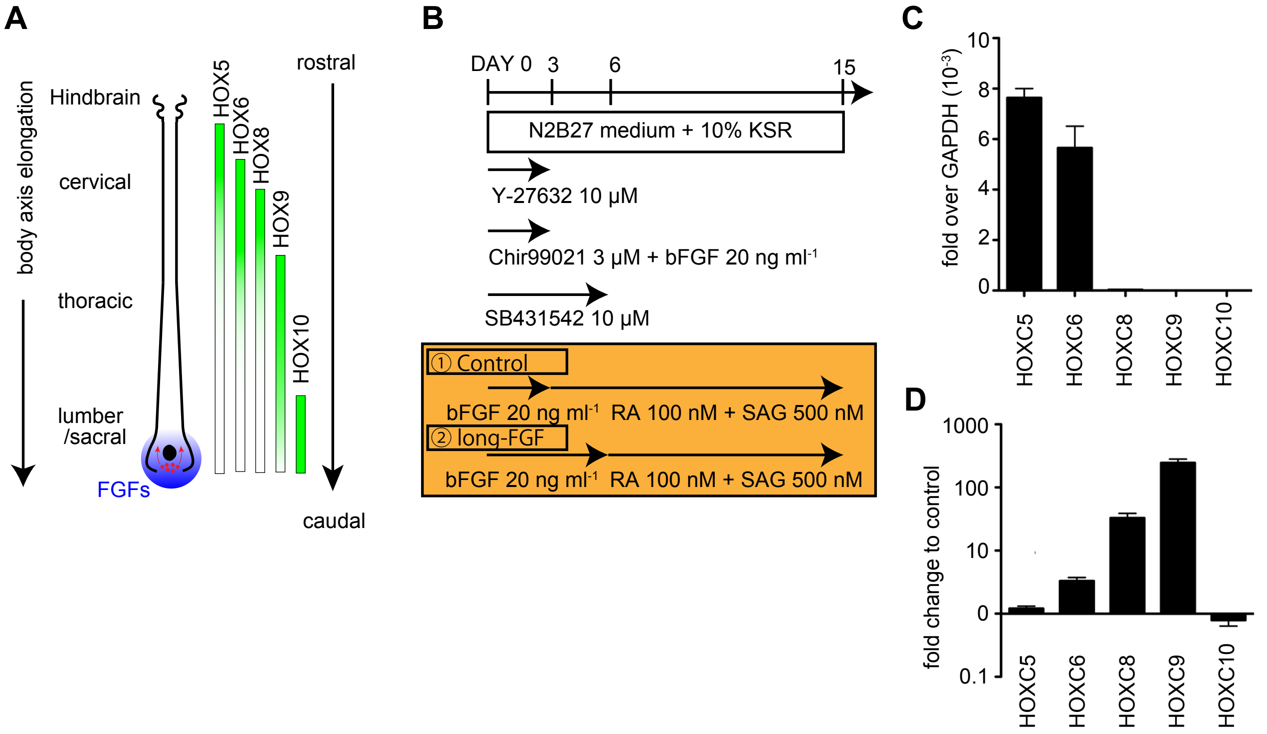

Fig. S7. Evaluation of positional identities along rostro-caudal axis. (A) Schematic showing the expression pattern of HOX gene along the R-C axis. (B) Schematic of the examined conditions. (C) qPCR analysis on day 15 showing the expression of HOX genes under control conditions (n=5 total RNA samples from 3 independent culture experiments) (D) qPCR analysis on day 15 showing the relative expression of HOX genes under long FGF treatment conditions compared to control conditions (n=5 total RNA samples from 3 independent culture experiments). HOXC8 and HOXC9 were uplegulated, while HOXC10 was not. Each bar represents s.e.m.

Development • Supplementary information

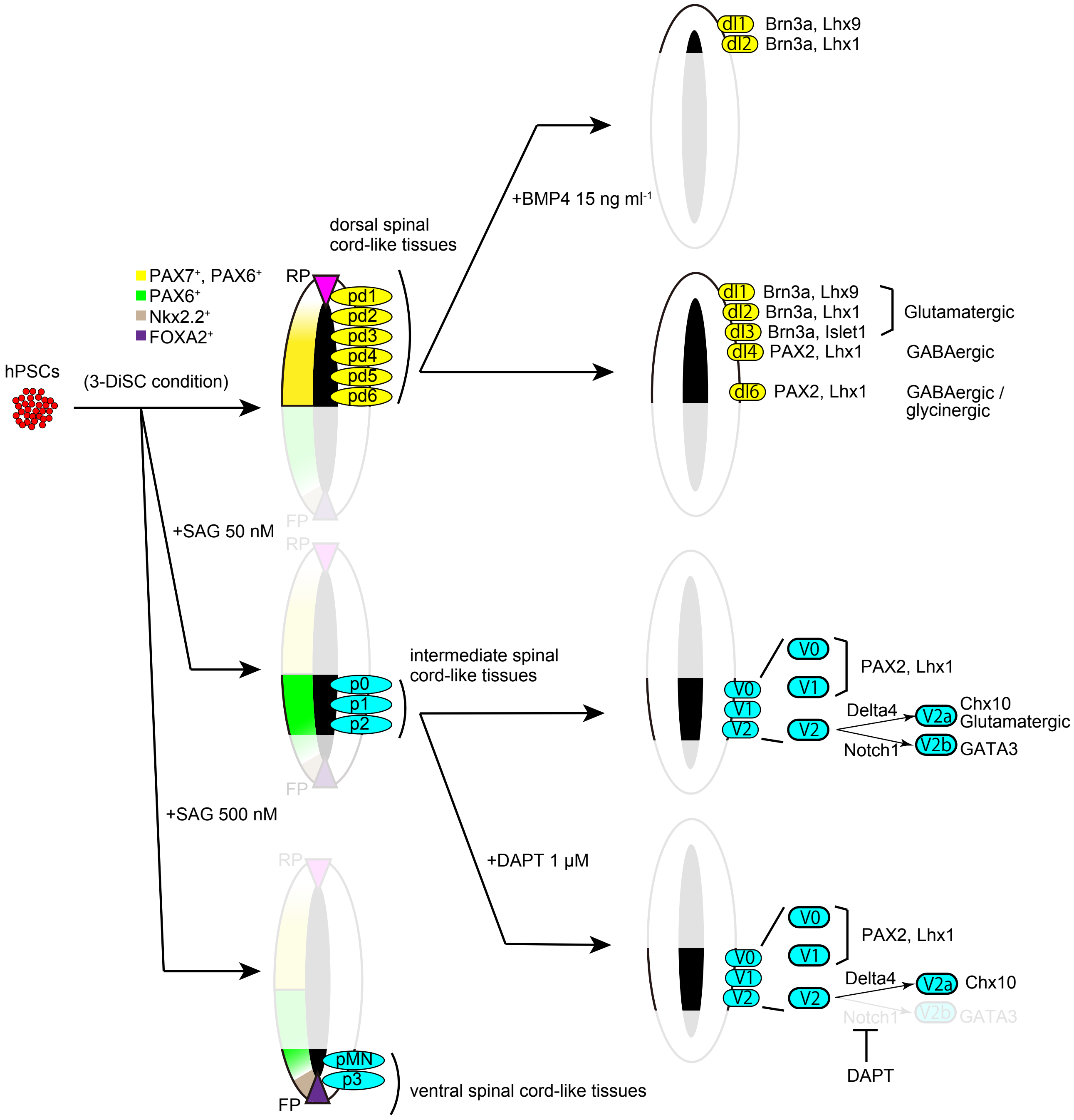

Fig. S8. Schematic summary of examined conditions for 3D spinal cord tissues.

Development • Supplementary information

Table. S1. List of primary antibodies and their dilutions for Immunostaining

Antibodies	Company	Catalog#	Host spices	Dilution
Arx	R&D	AF7068	Sheep	1:100
PKCζ	SantaCruz	sc-216	Rabbit	1:100
Brachyury	R&D	AF2085	Goat	1:200
Brn3a	SantaCruz	sc-31984	Goat	1:250
Calbindin	Swant	D-28K	Mouse	1:1000
ChAT	Millipore	AB144P	Goat	1:200
Chx10	Santa Cruz	sc-21690	Goat	1:500
Dbx1	Sigma	HPA039802	Rabbit	1:100
Evx1	DSHB	99.1-3A2	Mouse	1:100
FOXA2	Santa Cruz	sc-6554	Goat	1:500
FOXP1	Abcam	ab32010	Mouse	1:500
FOXP2	Abcam	ab16046	Rabbit	1:4000
GABA	Millipore	MAB316	Mouse	1:250
GABA	Sigma	A2052	Rabbit	1:2000
GAD67	Millipore	MAB5406	Mouse	1:1000
GATA3	R&D	MAB6330	Mouse	1:500
GFAP	Santa Cruz	sc-6170	Goat	1:500
Hb9	DSHB	81.5C10	Mouse	1:100
Islet1	Sigma	HPA057416	Rabbit	1:500
Lhx1	DSHB	4F2	Mouse	1:100
Lhx9	Sigma	HPA009695	Rabbit	1:100
Lmx1a	Sigma	HPA030088	Rabbit	1:2000
MafB	Sigma	HPA005653	Rabbit	1:250
MSX1/2	DSHB	4G1	Mouse	1:200
N-cadherin	BD	610920	Mouse	1:1000
Nkx2.2	DSHB	74.5A5	Mouse	1:100
Nkx6.1	DSHB	F55A12	Mouse	1:100
Olig2	Millipore	AB9610	Rabbit	1:1000
Olig3	Sigma	HPA018303	Rabbit	1:100
PAX2	Covance	PRB-276P	Rabbit	1:500
PAX6	BD	561462	Mouse	1:500
PAX6	Covance	PRB-278P	Rabbit	1:500
PAX7	DSHB	PAX7	Mouse	1:100
phospho-SMAD1/5	Cell Signaling	9516	Rabbit	1:500
SOX1	R&D	AF3369	Goat	1:1000
SOX2	SantaCruz	sc-17320	Goat	1:500
SOX2	Abcam	ab97959	Rabbit	1:500
SOX10	Abcam	ab108408	Rabbit	1:500
Tuj1	Tuj1	MMS435P	Mouse	1:1000
Tuj1	Tuj1	PRB-435P	Rabbit	1:1000
Vglut2	R&D	MAB5504	Mouse	1:200
Vglut2	Synaptic Systems	135 404	Guinea Pig	1:1000
Wnt1	Abcam	ab15251	Rabbit	1:500

Development • Supplementary information

Table. S2. List of genes and their primer sequences for qPCR analysis

Gene	Forward (5’ -3’)	Reverse (5’ -3’)
Brn3a	CTCCCTGAGCACAAGTACCC	TGAAAGGATGGCTCTTGCCC
Dbx1	GTACATCAGCAAGCCCGACC	AGTTCCGCCATTTCATGCGT
Dbx2	CCCAACAGCACTCAAGTCCA	AGCCCCAGTAAGTACACCCT
FOXA2	TTCAGGCCCGGCTAACTCT	AGTCTCGACCCCCACTTGCT
HOXC5	GAGGAGCGAGCTAAGAGCAG	GTGGCTCATGTGCAGTTTGG
HOXC6	ACAGACCTCAATCGCTCAGG	GTACCGCGAGTAGATCTGGC
HOXC8	CCTCCGCCAACACTAACAGT	AAACATGAGGCTGGGAGACG
HOXC9	CGTGGACTCGCTCATCTCTC	AAAATCGCTACAGTCCGGCA
HOXC10	AGTGACTTCAATTGCGGGGT	GATAGGTGTTGAGGGCGAGG
Lbx1	CGCCAGCAAGACGTTTAAG	CTGCCCAAAGATGGTCATAC
Lmx1a	TCAGAAGGGTGATGAGTTTGTCC	GGGGCGCTTATGGTCCTTG
Nkx2.2	AAACCATGTCACGCGCTCA	GGCGTTGTACTGCATGTGCT
Nkx6.1	CACACGAGACCCACTTTTTCC	CCCAACGAATAGGCCAAACG
Olig2	GGGCCACAAGTTAGTTGGAA	GAGGAACGGCCACAGTTCTA
Olig3	CCTGCTCGCCAGAAACTACA	CCCCATAGATCTCGCCAACC
PAX6	TCTTTGCTTGGGAAATCCG	CTGCCCGTTCAACATCCTTAG
PAX7	CTTCAGTGGGAGGTCAGGTT	CAAACACAGCATCGACGG
GAPDH	GGTCGGAGTCAACGGATTTG	TCAGCCTTGACGGTGCCATG

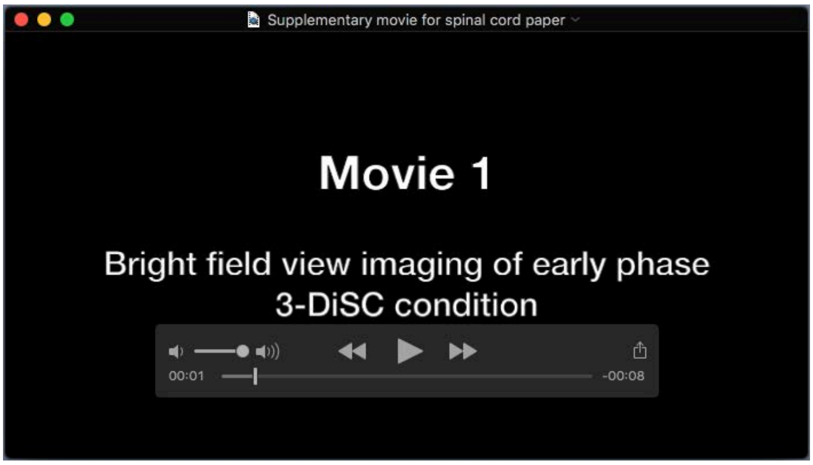

Movie 1. Bright field view imaging of early phase 3-DiSC condition
The original imaging data were taken by using the IncuCyte S3 Live Cell Imaging System every 2 hours for 66 hours during tissue differentiation under 3-DiSC condition. After seeding hPSCs to each well of a U-bottomed 96-well plate, a cell aggregate became obvious from 10 hours after the start of differentiation. Some cells were not included in the aggregate and remained around the aggregate. Scale bar: 400 µm.

Development • Supplementary information
